# Supplementary material for: Superhydrophobic Soot Coated Quartz Crystal Microbalances: A Novel Platform for Human Spermatozoa Quality Assessment
Source: Sensors (Basel). 2019 Jan 2;19(1):123. doi: 10.3390/s19010123 (PMC6338977; doi:10.3390/s19010123)
Supplement: Supplementary file 1 [file sensors-19-00123-s001.pdf]

Superhydrophobic soot coated quartz crystal microbalances: A novel platform for human spermatozoa quality assessment

Supporting information

[a]Karekin D. Esmeryan*, [b]Rumiana Ganeva, [b]Georgi S. Stamenov and [b]Todor A. Chaushev

[a]Acoustoelectronics Laboratory, Georgi Nadjakov Institute of Solid State Physics, Bulgarian Academy of Sciences, 72, Tzarigradsko Chaussee Blvd., 1784 Sofia, Bulgaria

[b]Research Department, Nadezhda Women's Health Hospital, 3 "BlagaVest" Street, Sofia, Bulgaria

*Corresponding author: tel. +359 2 979 5811; email: karekin_esmerian@abv.bg;

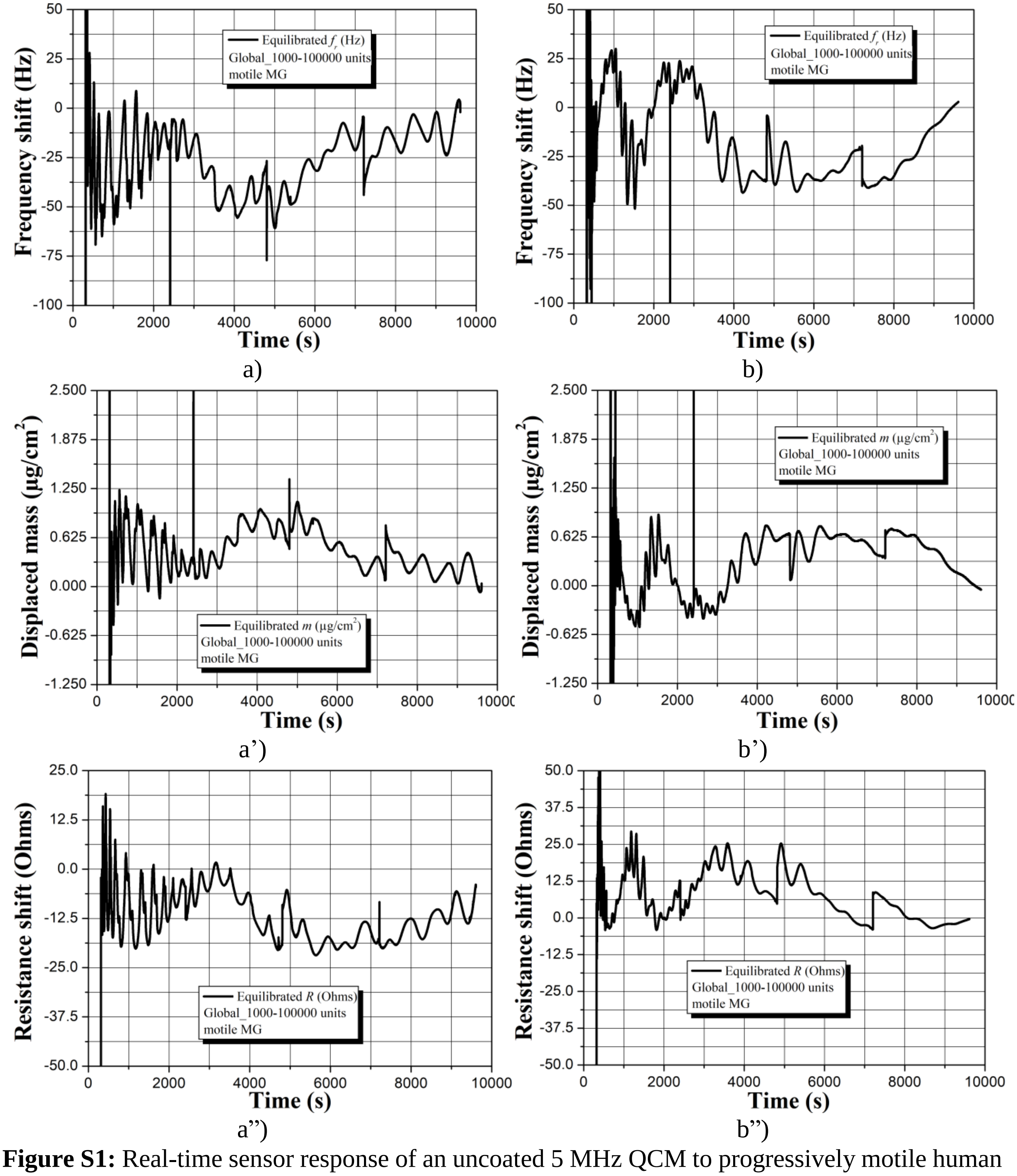

Figure S1: Real-time sensor response of an uncoated 5 MHz QCM to progressively motile human spermatozoa with gradually increasing concentration and its repeatability in two independent measurement cycles (a-a'' and b-b''). All graphs reflect actual biomass effects and/or possible viscosity-density changes after equilibration of the baselines in Global medium.

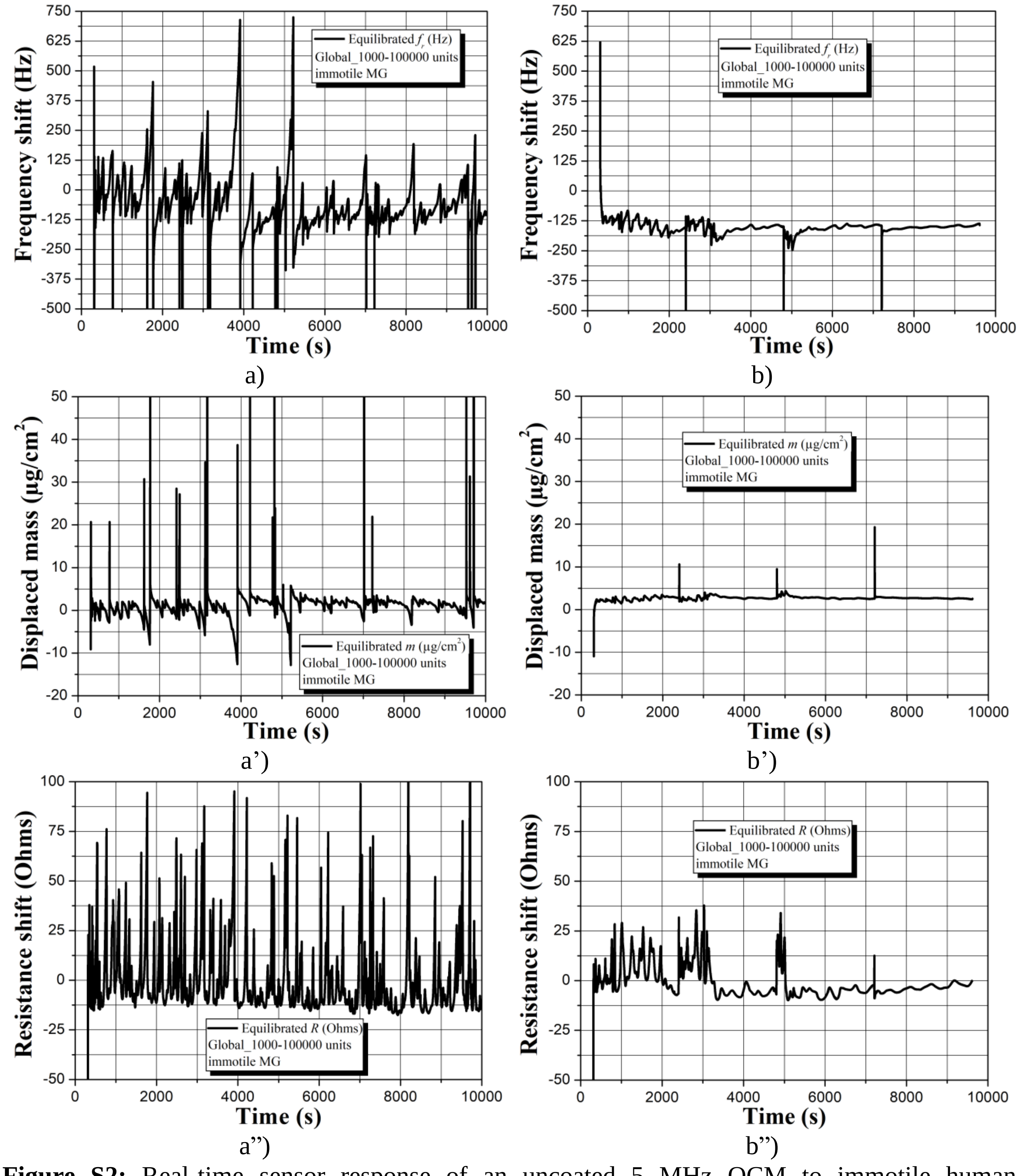

Figure S2: Real-time sensor response of an uncoated 5 MHz QCM to immotile human spermatozoa with gradually increasing concentration and its repeatability in two independent measurement cycles (a-a" and b-b"). All graphs reflect actual biomass effects and/or possible viscosity-density changes after equilibration of the baselines in Global medium.

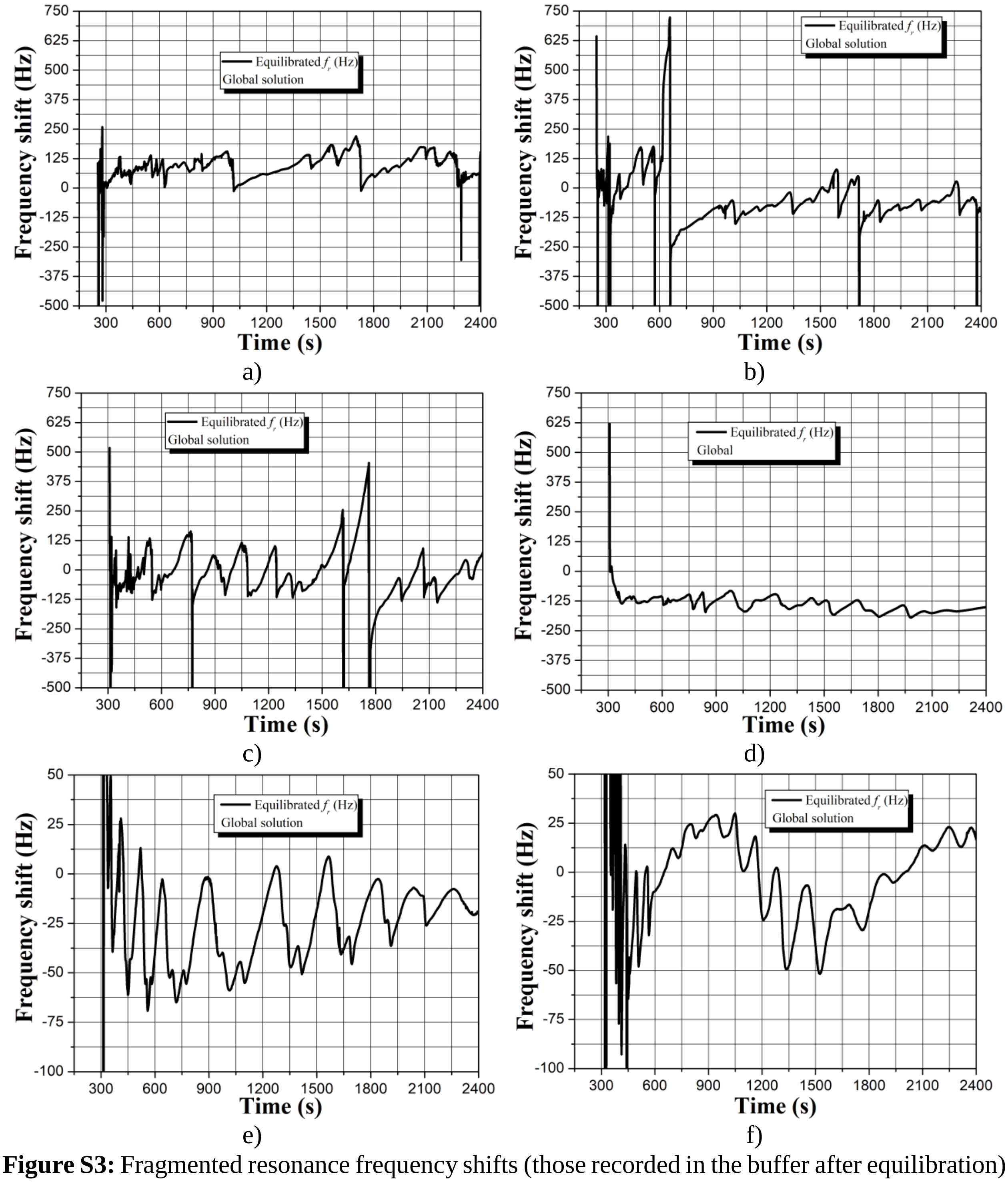

Figure S3: Fragmented resonance frequency shifts (those recorded in the buffer after equilibration) for an uncoated 5 MHz QCM before adding a) HSA and immotile spermatozoa at first measurement, b) HSA and immotile spermatozoa at second measurement, c) immotile spermatozoa at first measurement, d) immotile spermatozoa at second measurement, e) motile spermatozoa at first measurement and f) motile spermatozoa at second measurement.

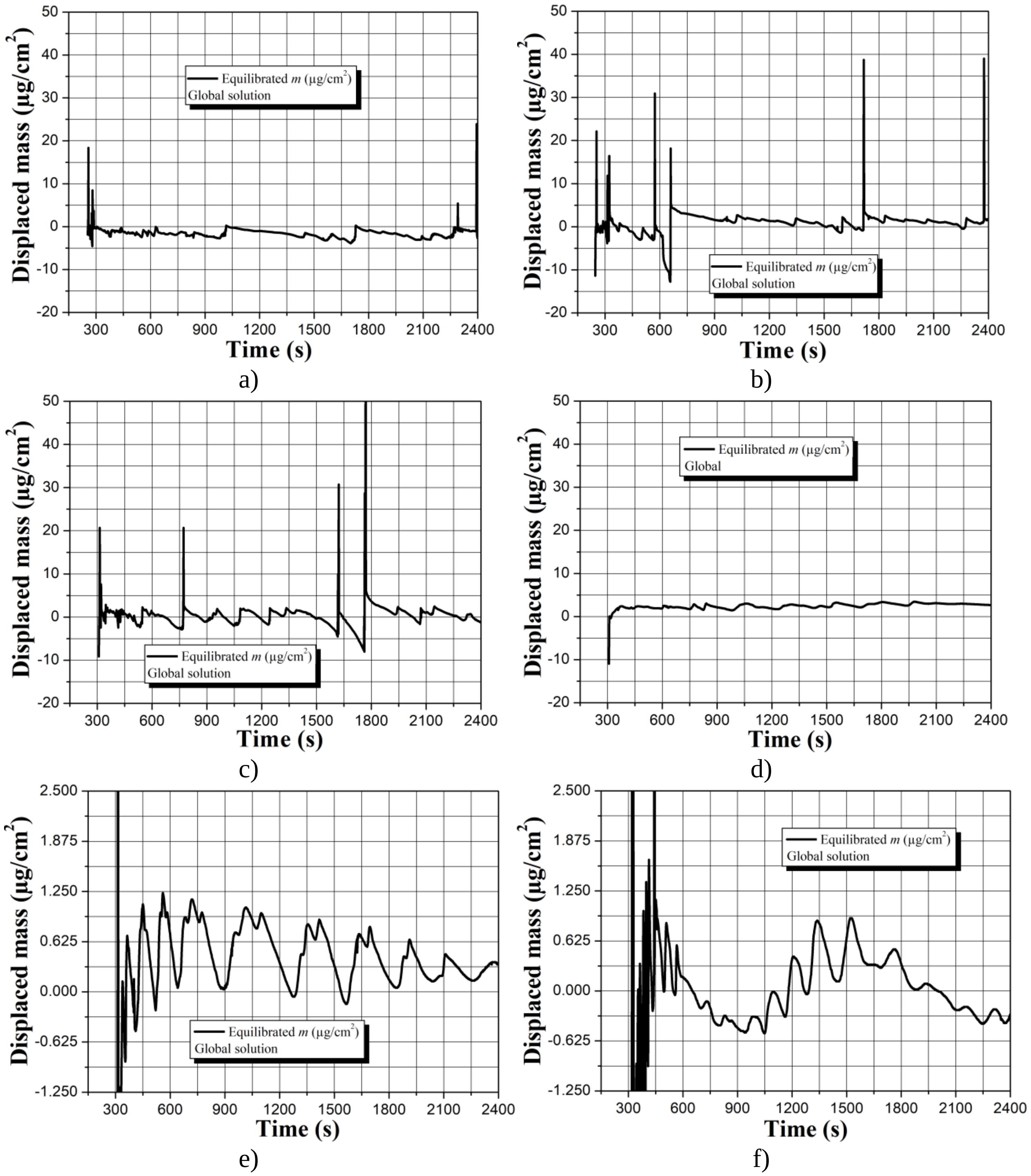

Figure S4: Fragmented mass displacement shifts (those recorded in the buffer after equilibration) for an uncoated 5 MHz QCM before adding a) HSA and immotile spermatozoa at first measurement, b) HSA and immotile spermatozoa at second measurement, c) immotile spermatozoa at first measurement, d) immotile spermatozoa at second measurement, e) motile spermatozoa at first measurement and f) motile spermatozoa at second measurement.

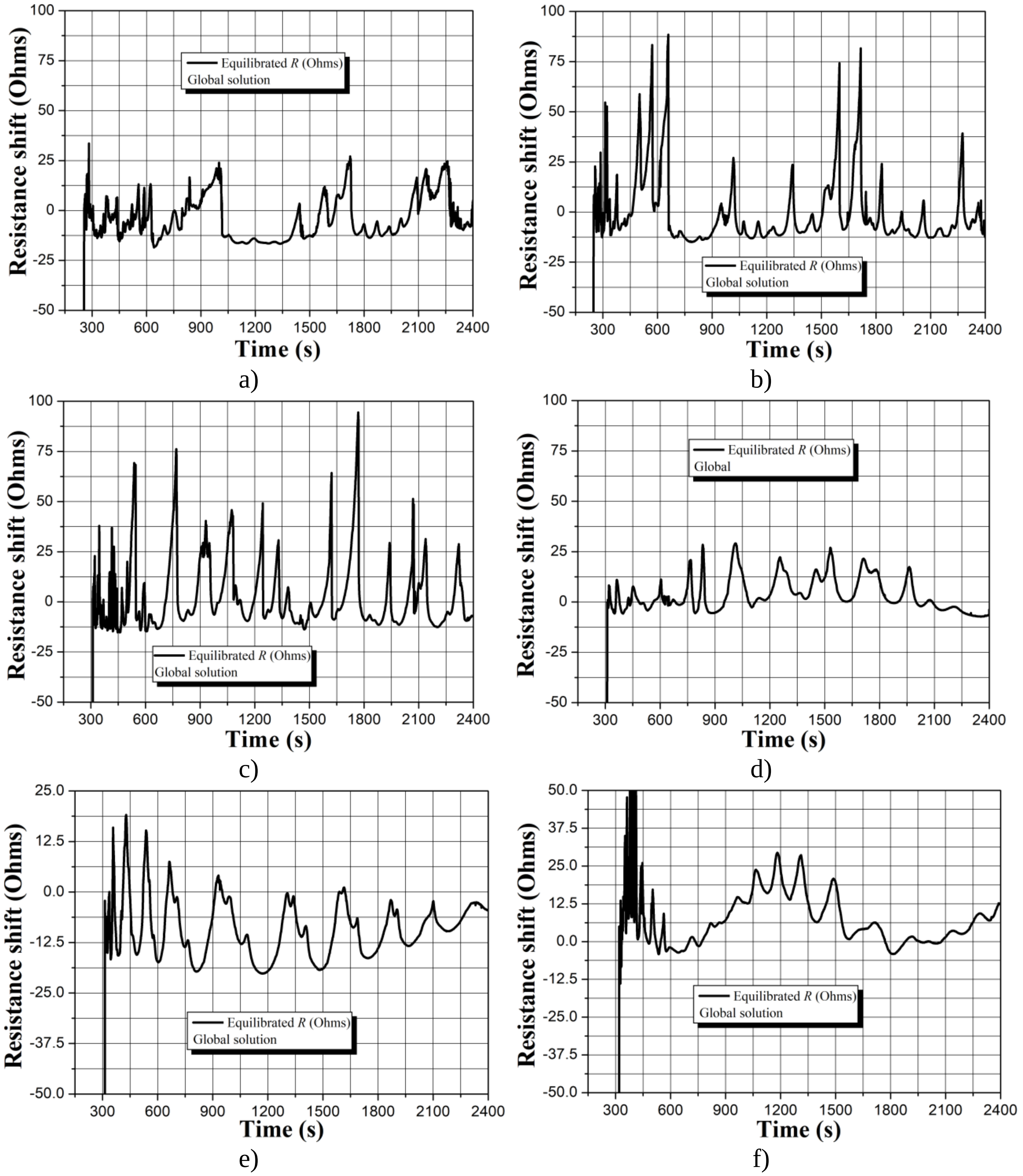

Figure S5: Fragmented dynamic resistance shifts (those recorded in the buffer after equilibration) for an uncoated 5 MHz QCM before adding a) HSA and immotile spermatozoa at first measurement, b) HSA and immotile spermatozoa at second measurement, c) immotile spermatozoa at first measurement, d) immotile spermatozoa at second measurement, e) motile spermatozoa at first measurement and f) motile spermatozoa at second measurement.

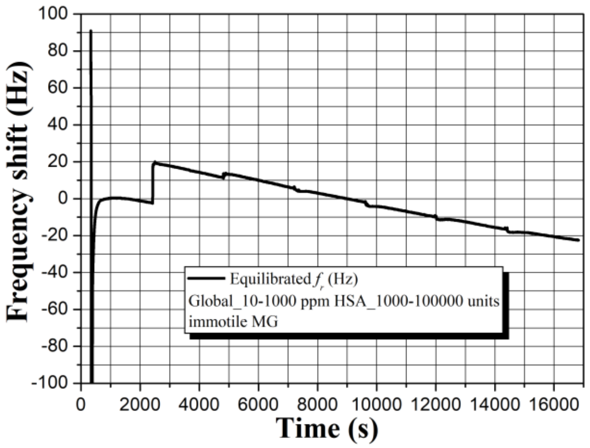

a)

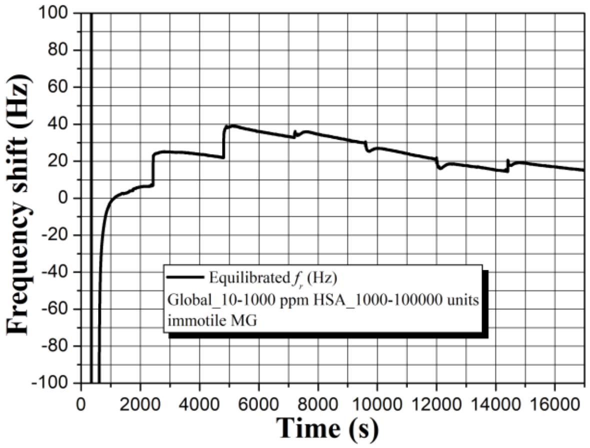

b)

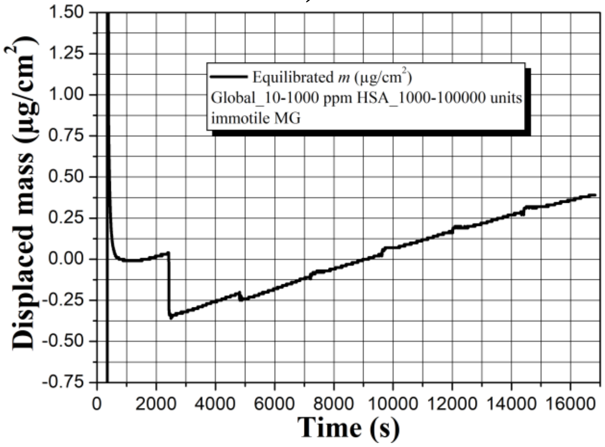

a’)

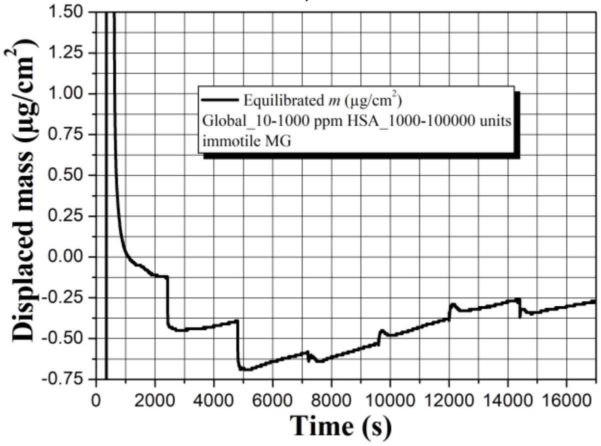

b’)

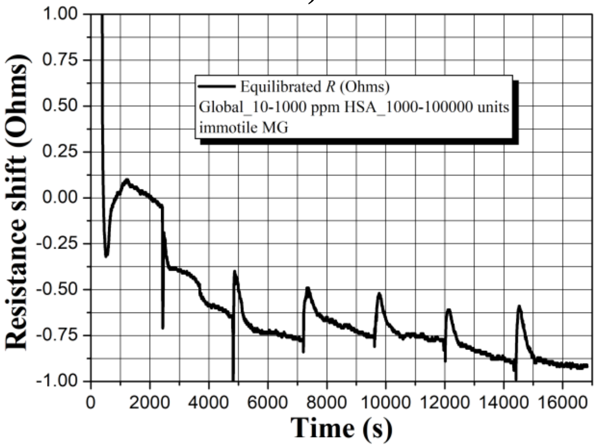

a”)

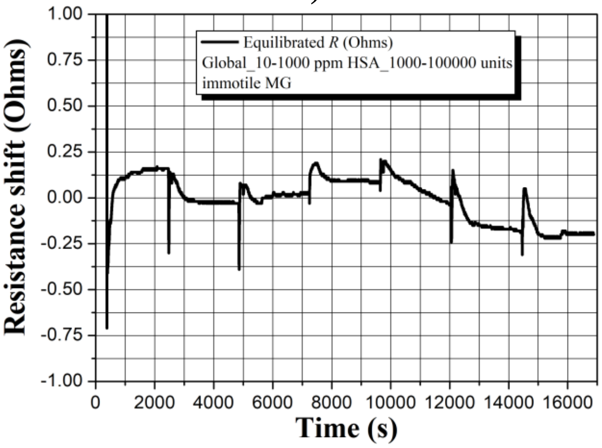

b”)

Figure S6: Real-time sensor response of a soot coated 5 MHz QCM to HSA and immotile human spermatozoa with gradually increasing concentration and its repeatability in two independent measurement cycles (a-a” and b-b”). All graphs reflect actual biomass effects after equilibration of the baselines in Global medium.

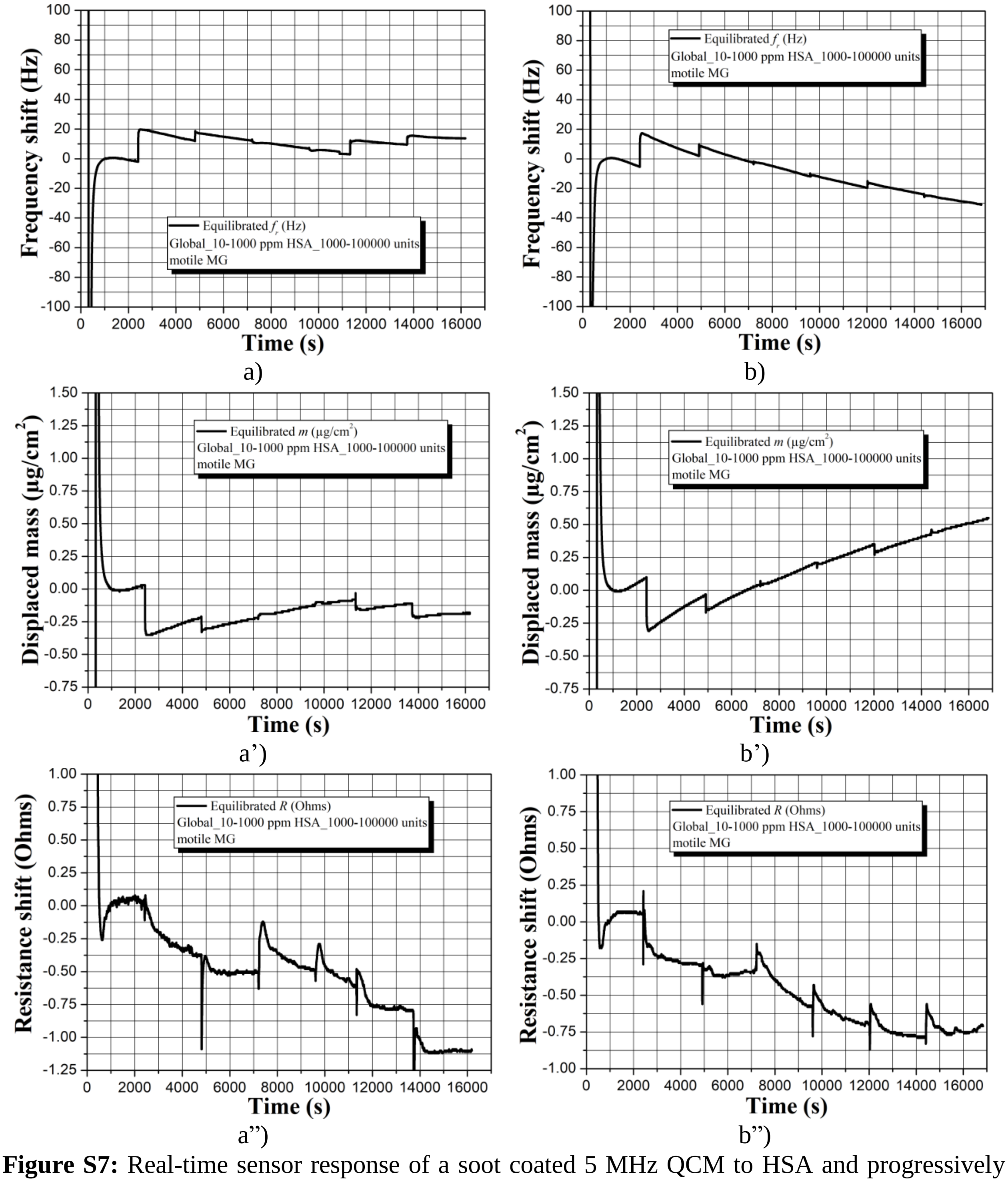

Figure S7: Real-time sensor response of a soot coated 5 MHz QCM to HSA and progressively motile human spermatozoa with gradually increasing concentration and its repeatability in two independent measurement cycles (a-a" and b-b"). All graphs reflect actual biomass effects after equilibration of the baselines in Global medium.

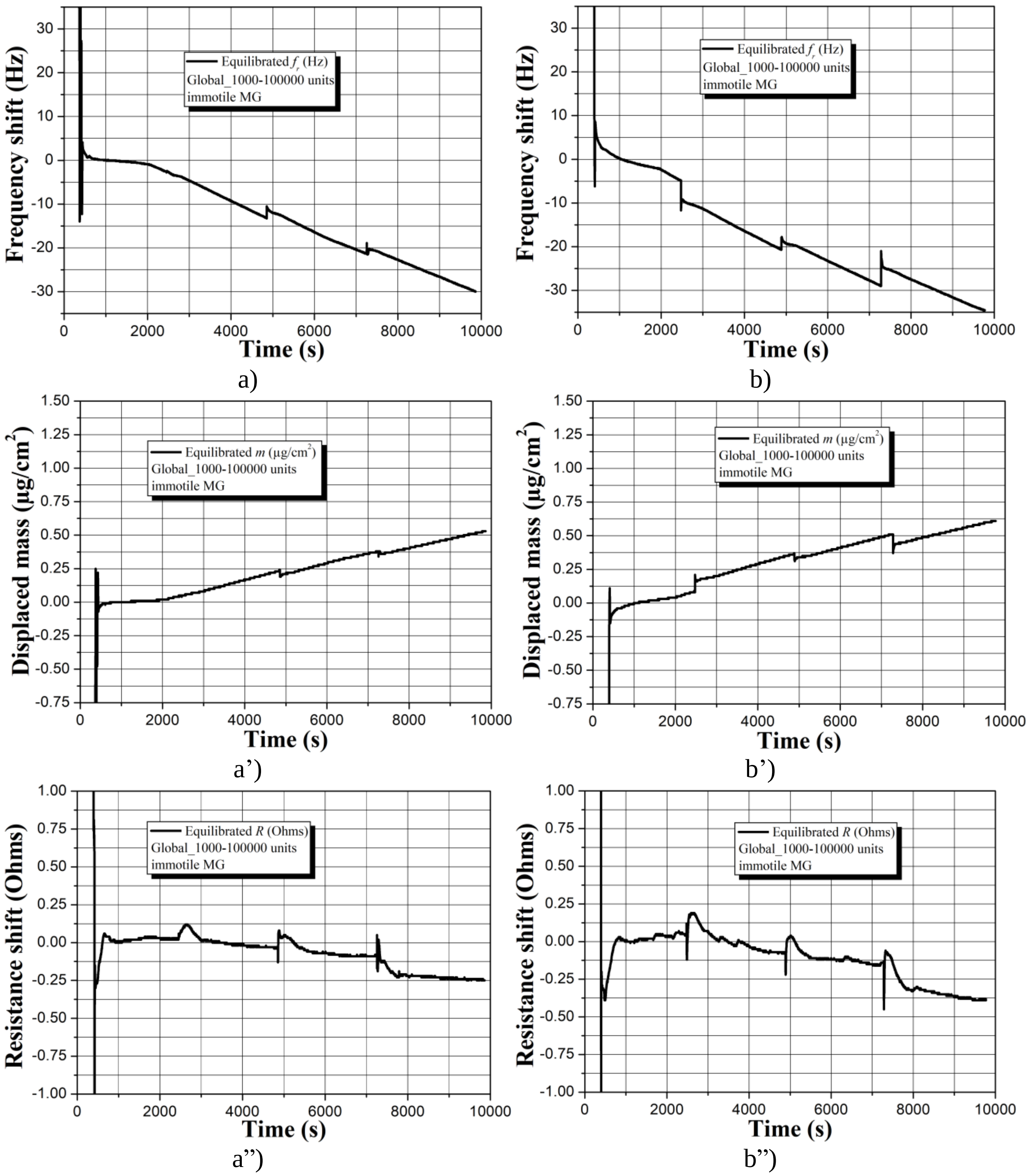

Figure S8: Real-time sensor response of a soot coated 5 MHz QCM to immotile human spermatozoa with gradually increasing concentration and its repeatability in two independent measurement cycles (a-a" and b-b"). All graphs reflect actual biomass effects after equilibration of the baselines in Global medium.

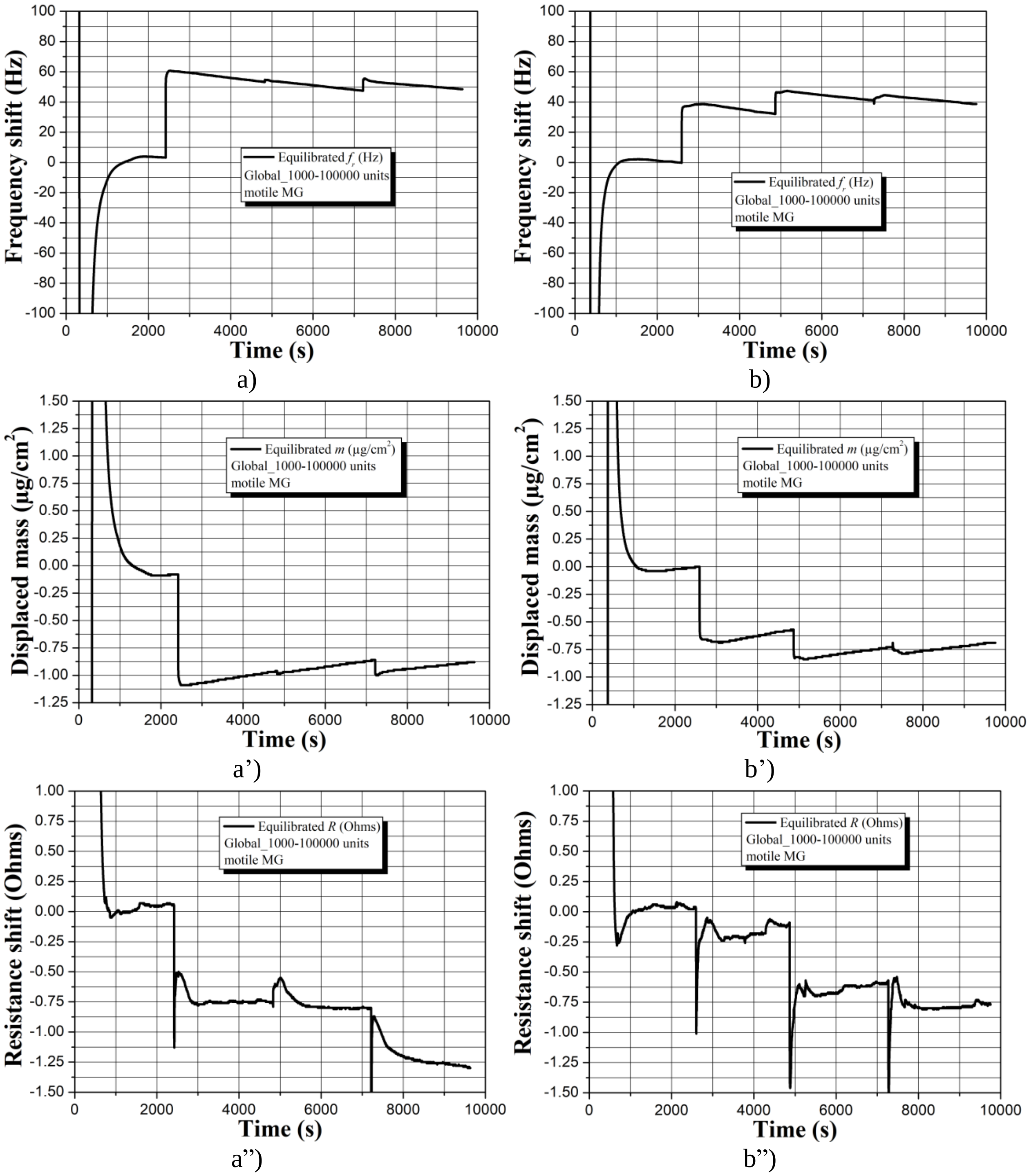

Figure S9: Real-time sensor response of a soot coated 5 MHz QCM to motile human spermatozoa with gradually increasing concentration and its repeatability in two independent measurement cycles (a-a" and b-b"). All graphs reflect actual biomass effects after equilibration of the baselines in Global medium.

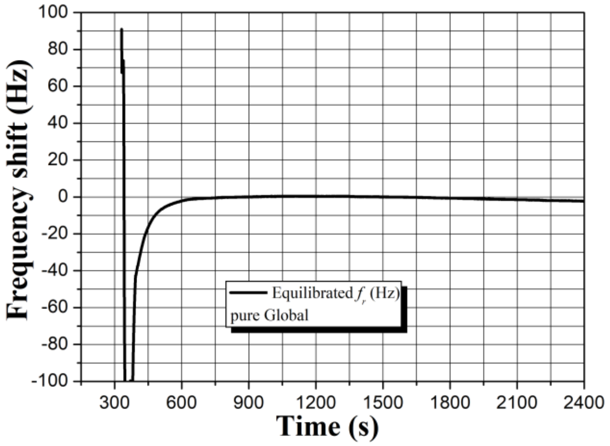
Frequency shift (Hz)
Time (s)
Equilibrated f_r (Hz)
pure Global

a)

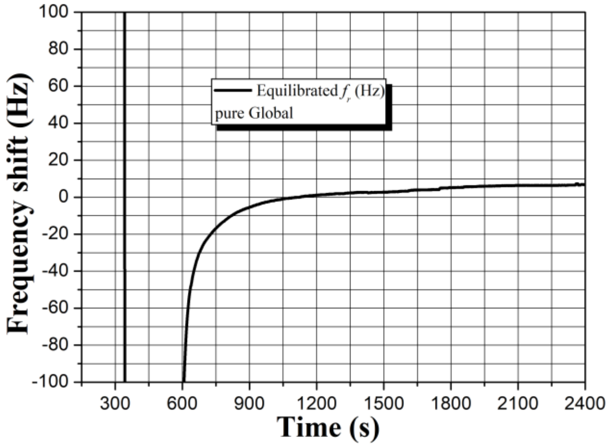
Frequency shift (Hz)
Time (s)
Equilibrated f_r (Hz)
pure Global

b)

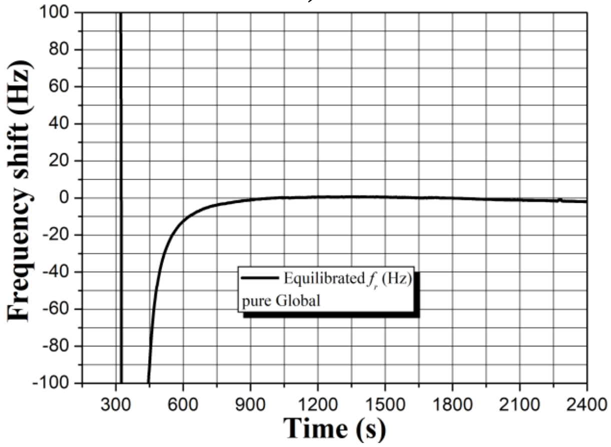
Frequency shift (Hz)
Time (s)
Equilibrated f_r (Hz)
pure Global

c)

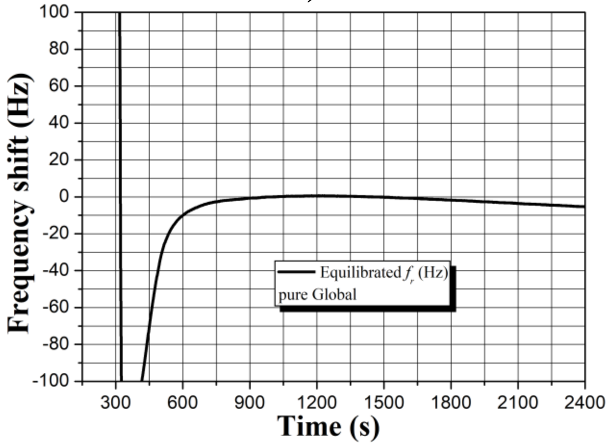
Frequency shift (Hz)
Time (s)
Equilibrated f_r (Hz)
pure Global

d)

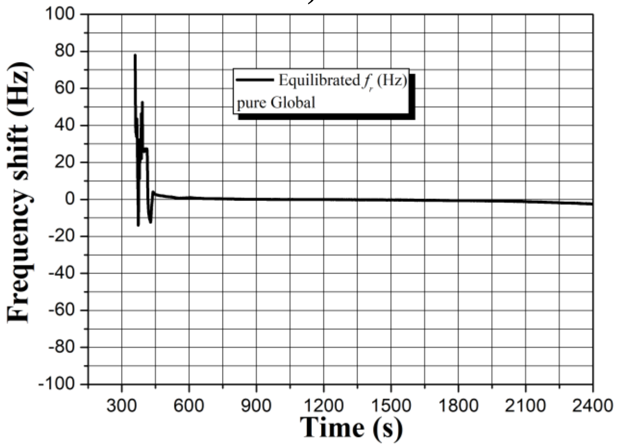
Frequency shift (Hz)
Time (s)
Equilibrated f_r (Hz)
pure Global

e)

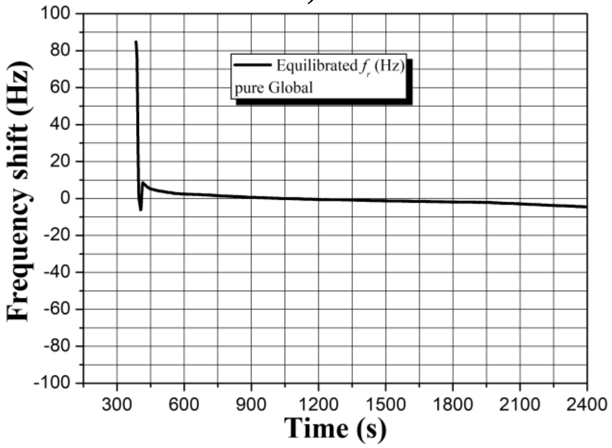
Frequency shift (Hz)
Time (s)
Equilibrated f_r (Hz)
pure Global

f)

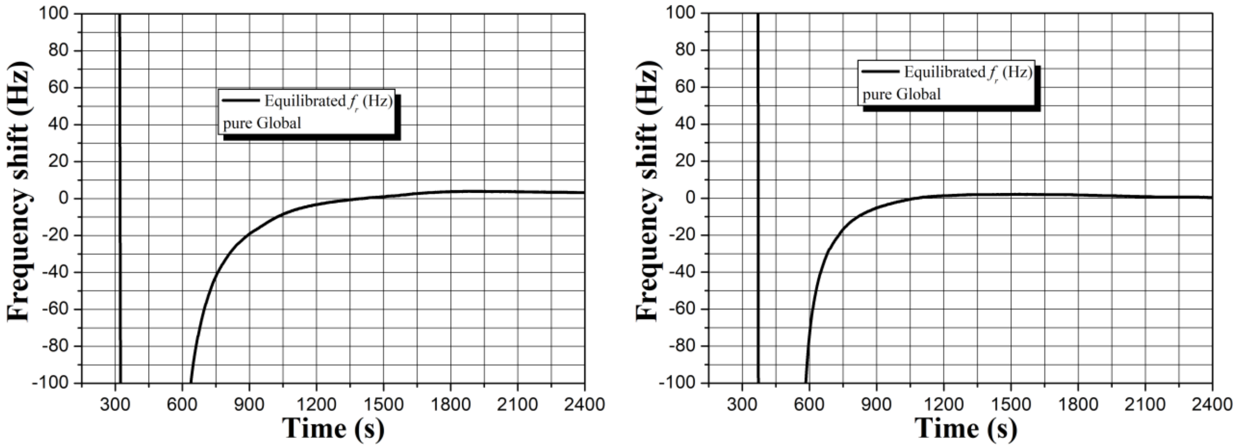

g) h)

Figure S10: Fragmented resonance frequency shifts (those recorded in the buffer after equilibration) for a soot coated 5 MHz QCM before adding a) HSA and immotile spermatozoa at first measurement, b) HSA and immotile spermatozoa at second measurement, c) HSA and motile spermatozoa at first measurement, d) HSA and motile spermatozoa at second measurement, e) immotile spermatozoa at first measurement and f) immotile spermatozoa at second measurement, g) motile spermatozoa at first measurement and h) motile spermatozoa at second measurement.

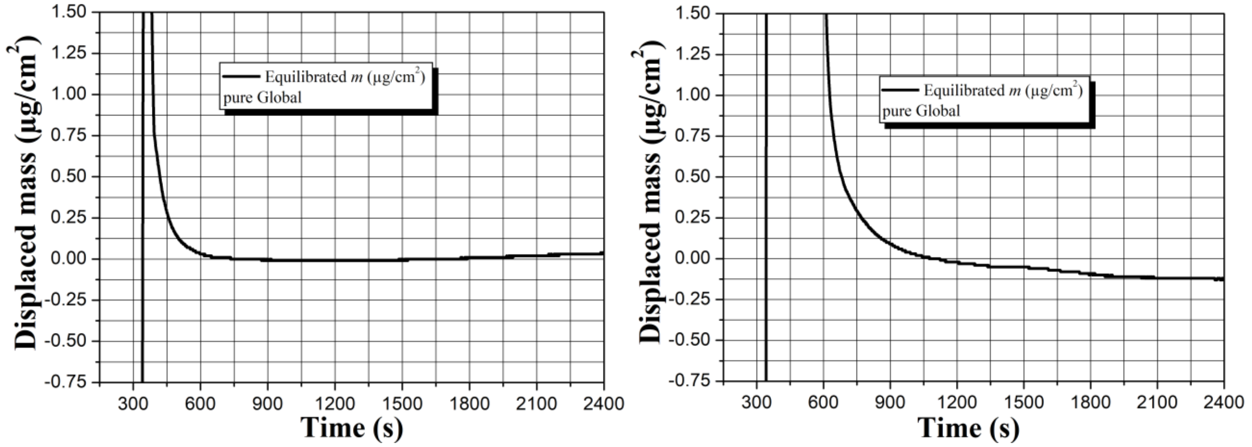

a) b)

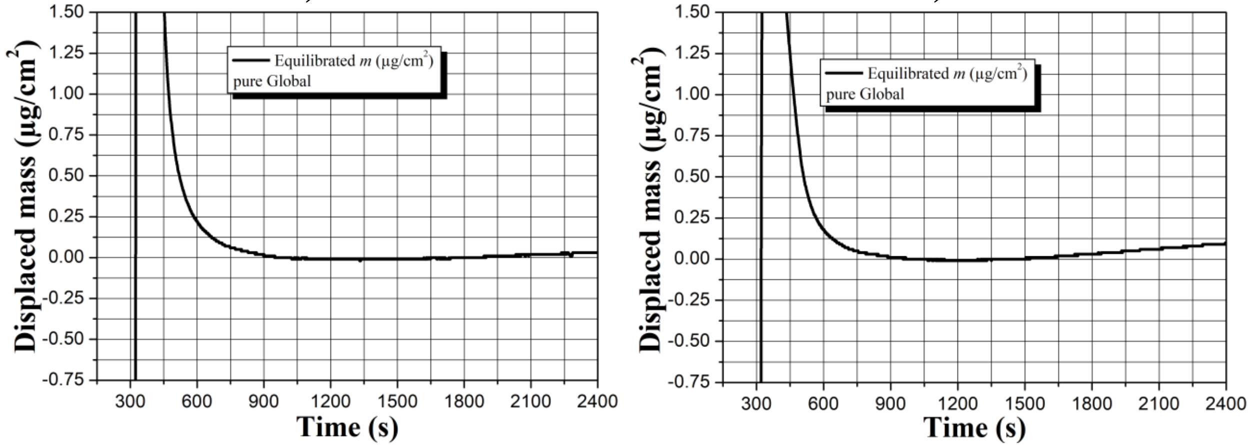

c) d)

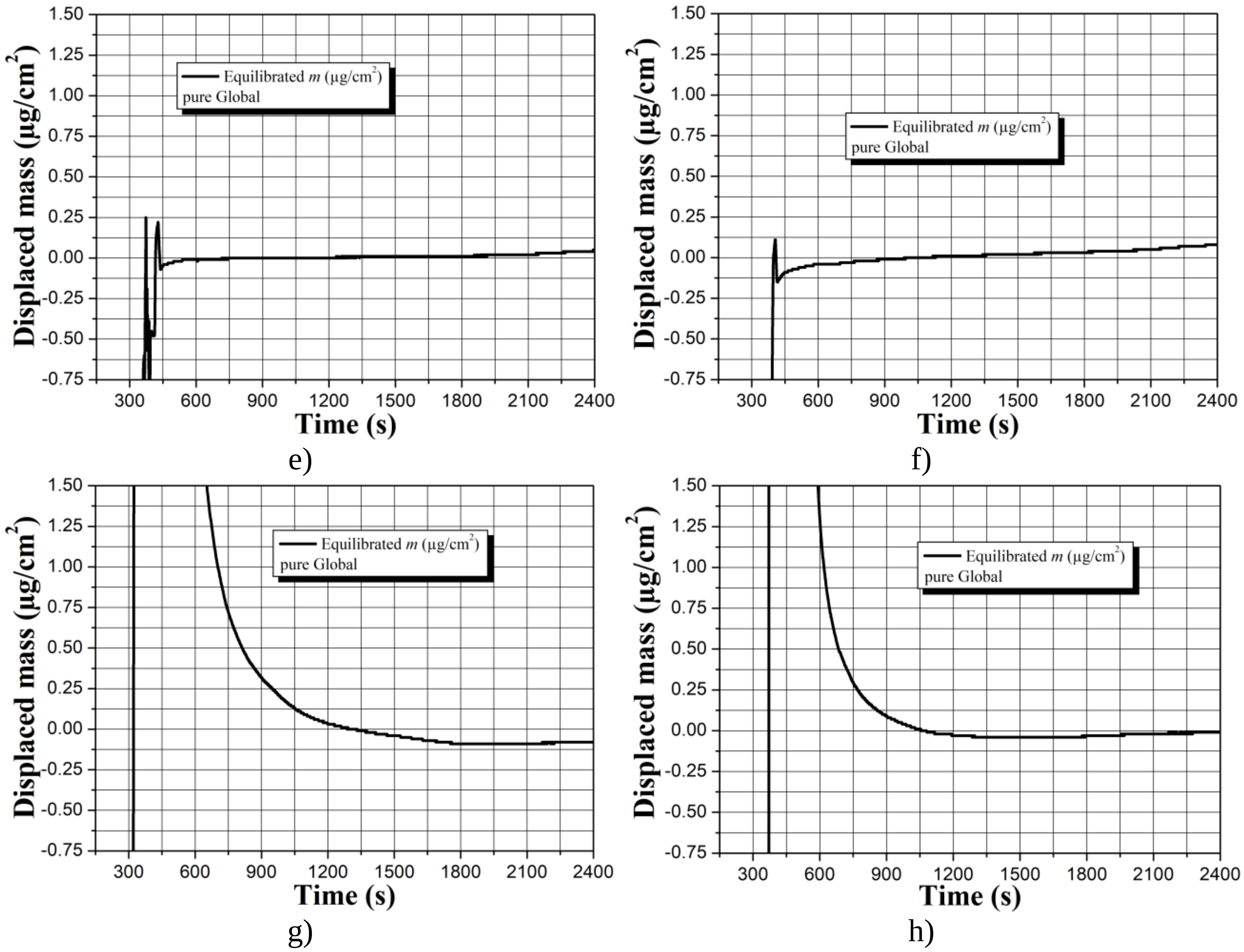

Figure S11: Fragmented mass displacement shifts (those recorded in the buffer after equilibration) for a soot coated 5 MHz QCM before adding a) HSA and immotile spermatozoa at first measurement, b) HSA and immotile spermatozoa at second measurement, c) HSA and motile spermatozoa at first measurement, d) HSA and motile spermatozoa at second measurement, e) immotile spermatozoa at first measurement and f) immotile spermatozoa at second measurement, g) motile spermatozoa at first measurement and h) motile spermatozoa at second measurement.

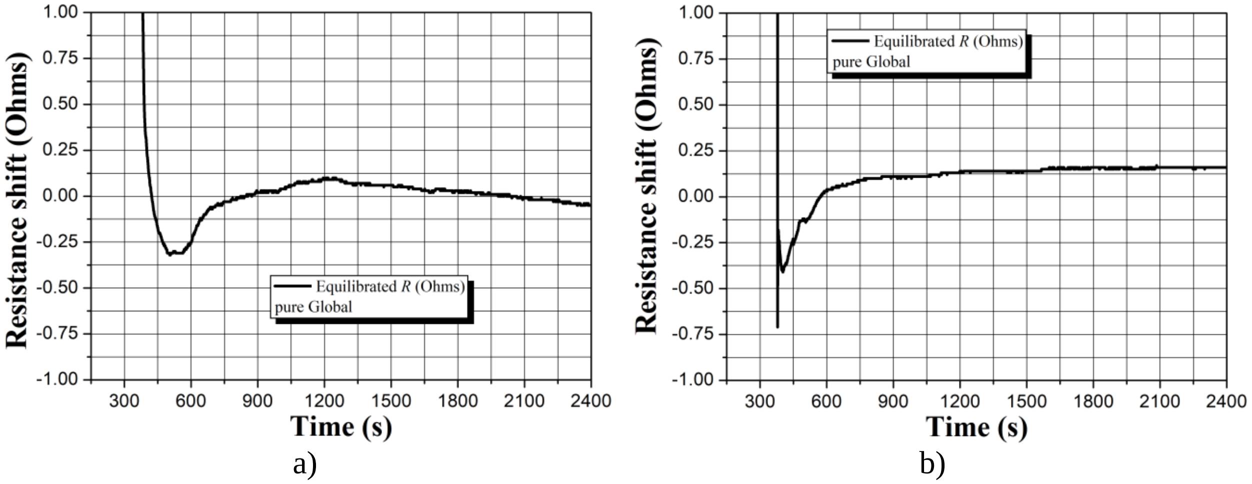

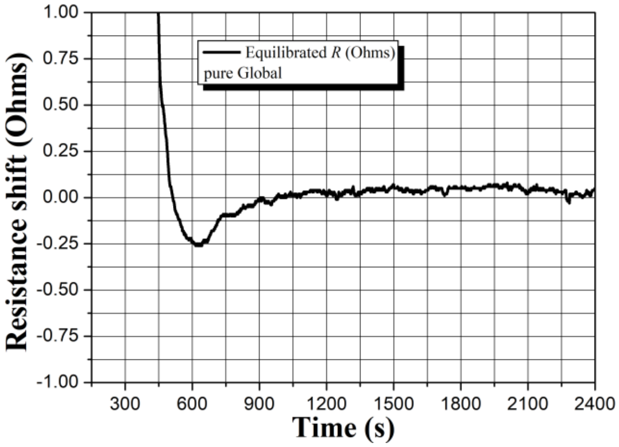

c)

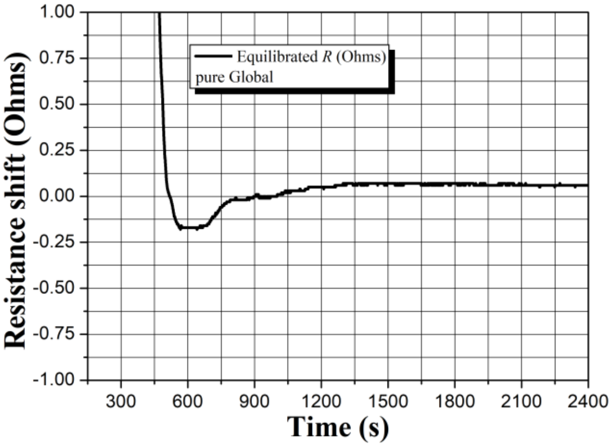

d)

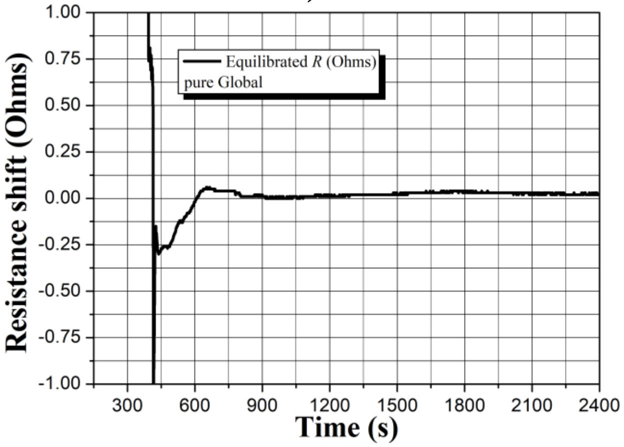

e)

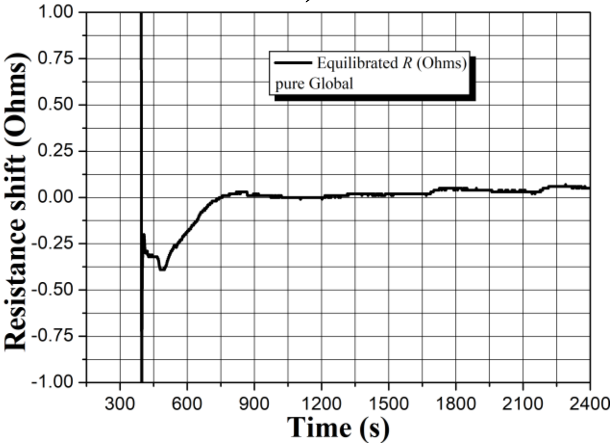

f)

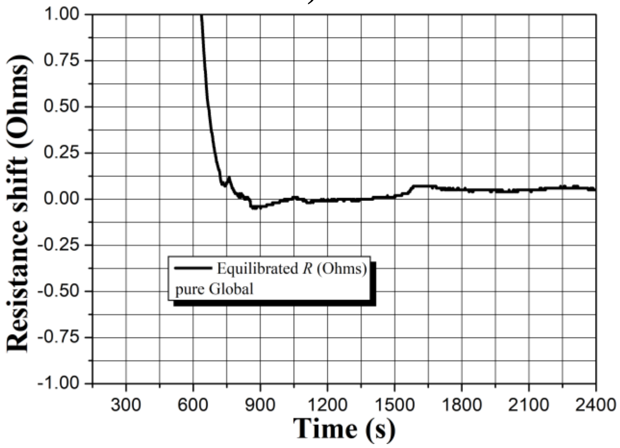

g)

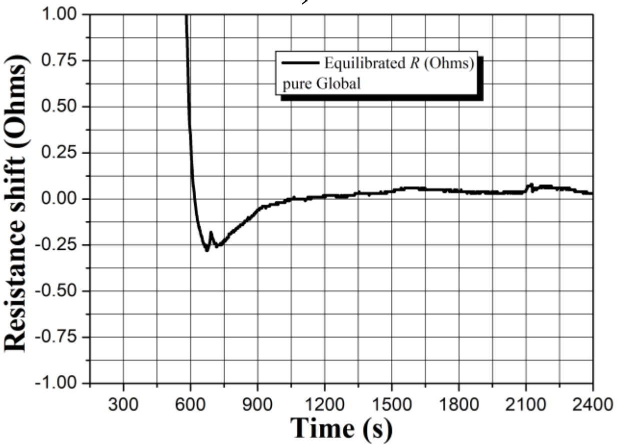

h)

Figure S12: Fragmented dynamic resistance shifts (those recorded in the buffer after equilibration) for a soot coated 5 MHz QCM before adding a) HSA and immotile spermatozoa at first measurement, b) HSA and immotile spermatozoa at second measurement, c) HSA and motile spermatozoa at first measurement, d) HSA and motile spermatozoa at second measurement, e) immotile spermatozoa at first measurement and f) immotile spermatozoa at second measurement, g) motile spermatozoa at first measurement and h) motile spermatozoa at second measurement.

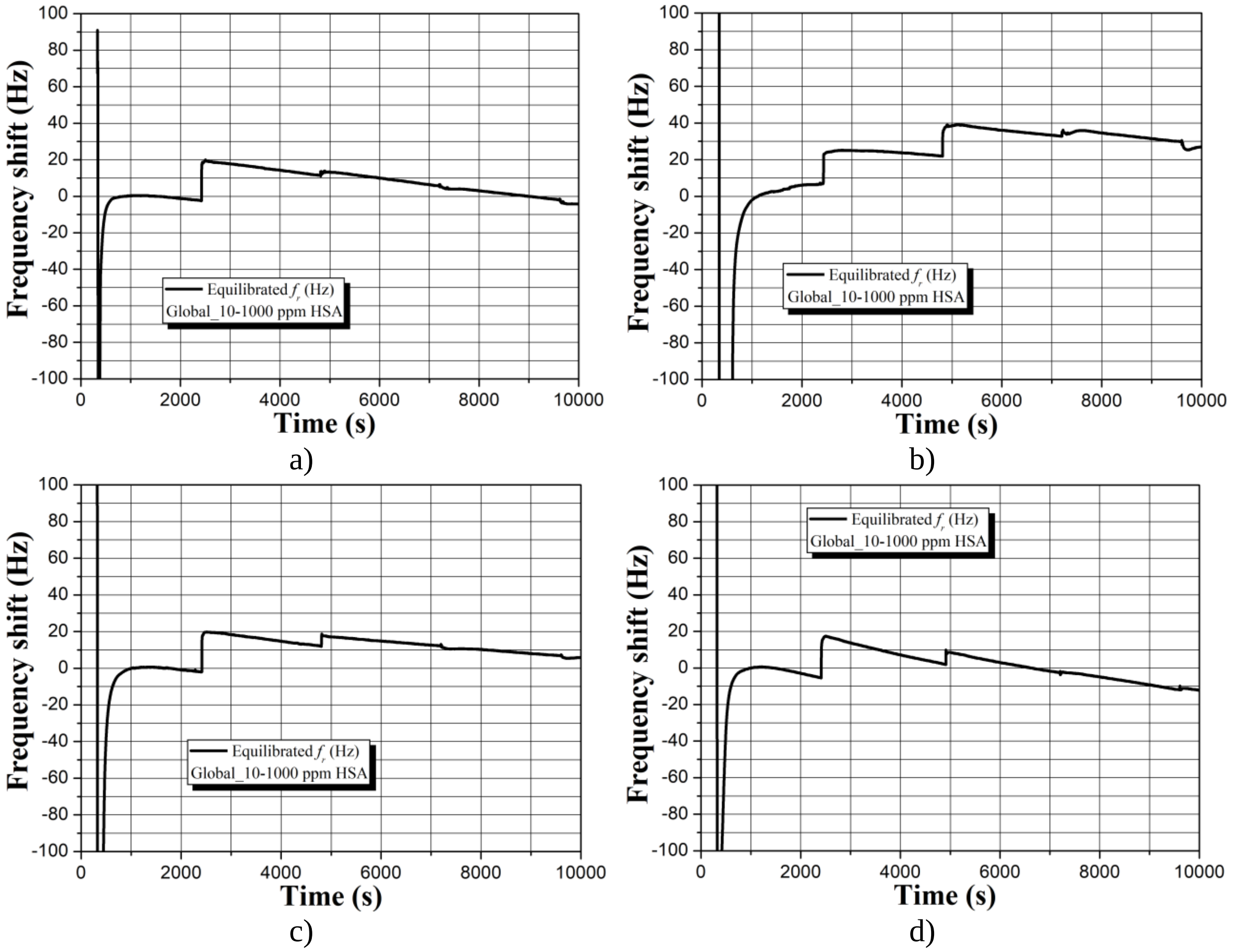

Figure S13: Resonance frequency shifts of a soot coated 5 MHz QCM to HSA with gradually increasing concentration before adding a) immotile spermatozoa at first measurement, b) immotile spermatozoa at second measurement, c) motile spermatozoa at first measurement and d) motile spermatozoa at second measurement.

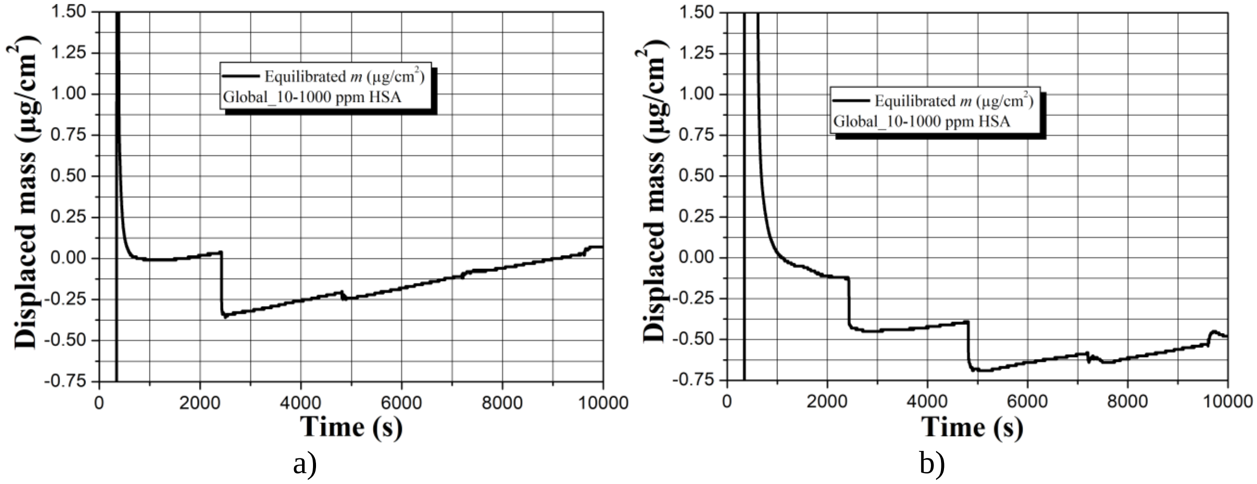

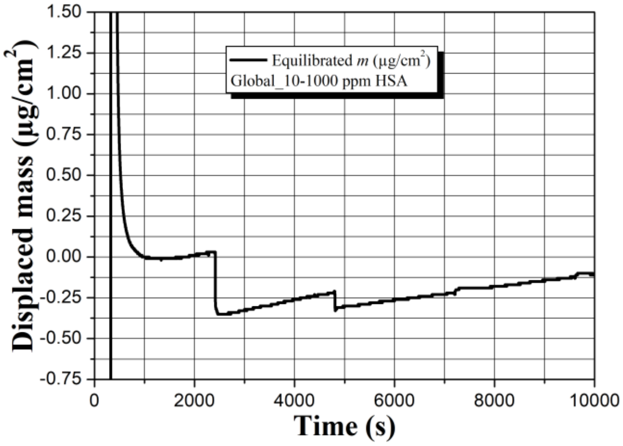

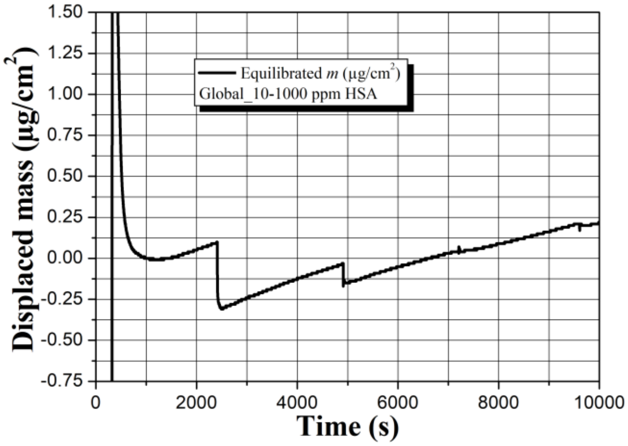

c) d)

Figure S14: Mass displacement shifts of a soot coated 5 MHz QCM to HSA with gradually increasing concentration before adding a) immotile spermatozoa at first measurement, b) immotile spermatozoa at second measurement, c) motile spermatozoa at first measurement and d) motile spermatozoa at second measurement.

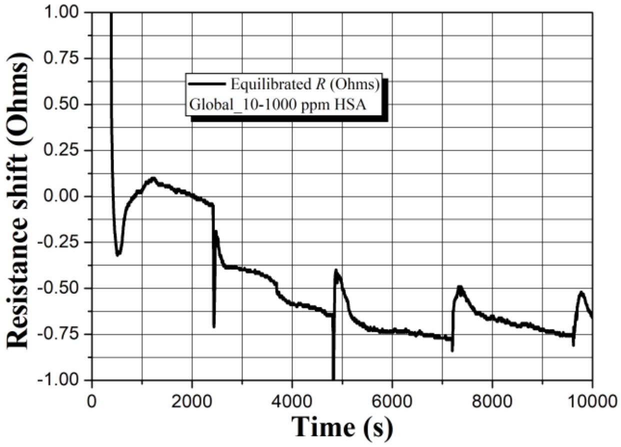

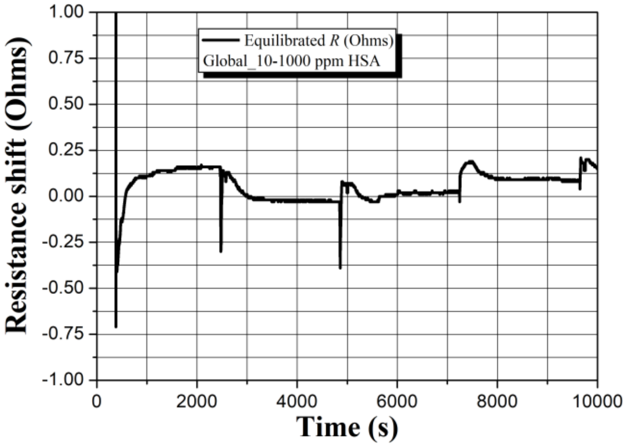

a) b)

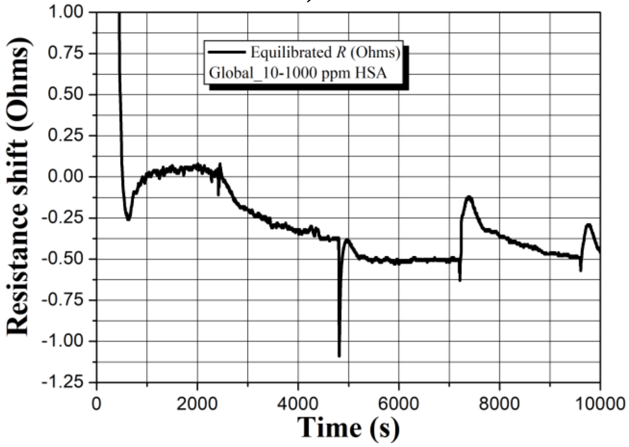

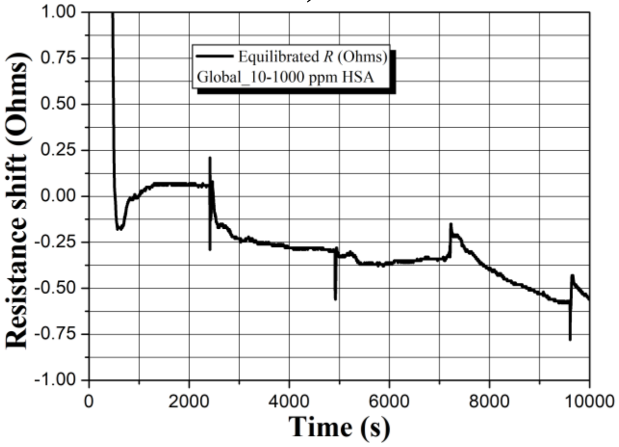

c) d)

Figure S15: Dynamic resistance shifts of a soot coated 5 MHz QCM to HSA with gradually increasing concentration before adding a) immotile spermatozoa at first measurement, b) immotile

spermatozoa at second measurement, c) motile spermatozoa at first measurement and d) motile spermatozoa at second measurement.
